# Correspondence

**Published:** 1894-05

**Authors:** W. E. Walker, Geo. B. Clement

**Affiliations:** Bay St. Louis, Miss.; Bay St. Louis, Miss.


### CORRESPONDENCE.

Bay St. Louis, Miss., April 7, 1894.

*Editor Dental Register:*

At the annual meeting of the Mississippi State Board of Dental Examiners, April 3, 4, 5, there were thirteen applicants, the successful candidates being J. R. Moore, W. P. Davis, W. O. Talbert, A. B. Kelly, A. T. Pierce and J. E. Johnson.

The system of the Illinois State Board, as recently published in the *Dental Review*, was adopted and worked very acceptably. No oral examinations were held, however, additional lists of questions being prepared on Oral Surgery, Prosthesis and Orthodontia. Each applicant who passed the written examination successfully was also required to clinic—the first time that this has been attempted by the Mississippi Board.

To secure the removal from the Board of A. H. Hilzem, a man who persisted in advertising contrary to the code of ethics (and who for this reason is no longer a member of the State Association) but who refused to resign from the Board, and whom the Governor declined to remove—but who consented to resign with the Board in a body—the entire Board resigned, each one pledging himself not to accept a re-appointment from the present Governor. This step was thought preferable to allowing a man setting such an evil example to the younger members of the profession, to occupy such a prominent position as that of member of the Board of Dental Examiners.

W. E. Walker, President.
Geo. B. Clement, Secretary.

